# A label-free electrochemical platform for the highly sensitive detection of hepatitis B virus DNA using graphene quantum dots

**DOI:** 10.1039/c7ra11945c

**Published:** 2018-01-08

**Authors:** Qian Xiang, Jingyun Huang, Huiyao Huang, Weiwei Mao, Zhizhen Ye

**Affiliations:** School of Materials Science and Engineering, Zhejiang University of China Hangzhou 310027 China huangjy@zju.edu.cn

## Abstract

Based on the strong interaction between single-stranded DNA and graphene material, we have designed a simple but smart electrochemical platform to detect HBV-DNA by using a graphene quantum dot (GQD) modified glassy carbon electrode coupled with specific sequence DNA molecules as probes. The probe DNA is designed to be complementary to the HBV-DNA, when the probe DNA is strongly bound to the surface of the GQD modified electrode the transfer of an electron from the electrode to the electrochemically active species K_3_[Fe(CN)_6_] will become difficult. Nevertheless, if the target HBV-DNA is found in the test solution, the probe DNA will bind with the target HBV-DNA instead of GQDs. As a result, the obtained peak currents of K_3_[Fe(CN)_6_] will have a different degree of increase with the different concentrations of the target HBV-DNA. In particular, the proposed sensor exhibits high sensitivity with a detection limit of 1 nM, and the linear detection range is from 10 nM to 500 nM. Additionally, the sensor could be used in detecting other probe DNA, which may have potential applications in the future.

## Abstract 2

Based on the interaction between single-stranded probe DNA and graphene quantum dots (GQDs), we have designed a simple but smart electrochemical platform to detect HBV-DNA by using GQDs modified glassy carbon electrode coupled with probe DNA.

## Graphical abstract

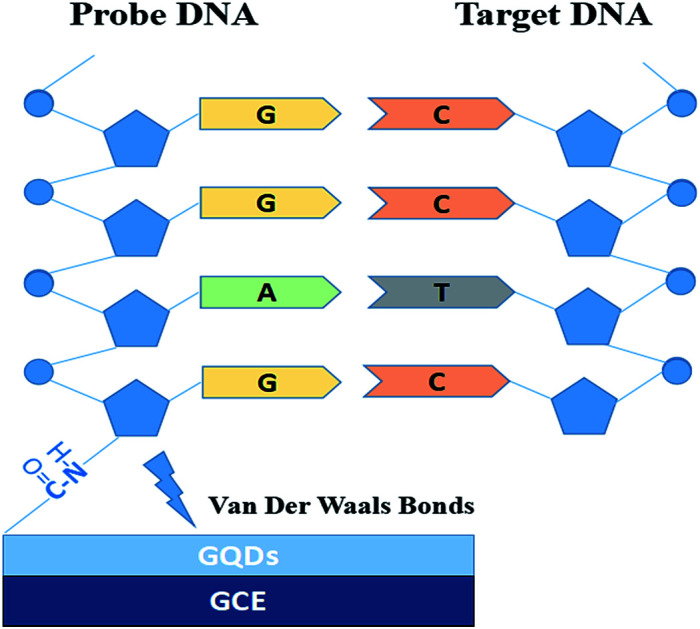

## 1. Introduction

As the main human pathogen, hepatitis B virus (HBV) is very harmful to humans and easily causes infection. There are surveys showing that there are 3 million HBV carriers worldwide. Liver inflammation is a common phenomenon in most patients, and some even get cirrhosis and liver cancer. At present, the most reliable and direct marker of HBV replication activity is HBV-DNA.^1^ As a result, in order to understand the degree of infection of patients with HBV and evaluate the efficacy of antiviral therapy, it is of great significance to make quantitative detection of HBV-DNA a realization. At present, many methods have been used to detect some specific genes or gene mutations, such as biological detection bar code technology, the signal amplification of DNA testing technology,^2^*etc.* Unfortunately, such methods have some drawbacks such as low sensitivity, needing expensive instruments or complex pretreatments, consumption of large sample volumes, time consuming, *etc.*^3^ In contrast, electrochemical methods have outstanding advantages including fast and low-cost, effective, simplicity, quantitative application and possibility of miniaturization. Consequently, it is helpful to find a simple, quick and sensitive electrochemical method to detect the DNA sequence of HBV.

Recently, based on their unique properties and excellent chemical sensing performances, a large number of nanomaterials have been used to prepare simple biological nanosensors. Graphene quantum dots (GQDs), since found in 2008 by Dai, it has become significant in research area as a new kind of carbon-based nanomaterial in recent years.^4^ It inherits the excellent properties of grapheme such as strength, large specific surface area.^5^ Combined with the advantages of quantum dots as the quantum confined effect, size effect and edge effect, GQDs also shows many fascinating properties, such as desirable biocompatibility, low cytotoxicity, excellent solubility, stable photoluminescence, thus, making them potential application in the areas of sensor systems and bio-imaging. At present, there are mainly two developed routes for GQDs synthesis, ‘top-down’ and ‘bottom-up’ methods. Top-down methods are different physical or chemical approaches to make bulk carbon materials into GQDs, including ionic liquid assisted grinding,^6^ hydrothermal,^7,8^ chemical ablation,^9^ photo-fenton oxidation,^10^ oxygen plasma treatment, electrochemical oxidation^11^ and *etc.* On the contrary, bottom-up methods mainly converse suitable organic precursors to GQDs by the ways such as microwave,^12^ solvothermal treatment,^13–15^ thermal pyrolysis,^16,17^*etc.* Hence, there are obvious advantages of bottom-up methods over top-down methods, because the composition and physical properties of GQDs can be easily adjusted by careful selecting precursors from diversified organic compounds as well as the carbonization conditions.^16,17^

In this contribution, an ultra-sensitive label-free electrochemical biosensor using GQDs for detecting HBV-DNA was made. GQDs was synthesized by a safe and simple bottom-up methods, and can be directly modified onto the surface of glassy carbon electrode (GCE) because of the physical adsorption with van der Waals forces.^18,19^ And then we chose K_3_[Fe(CN)_6_] as the electroactive indicator to detect and monitor what changes were happening on the surface of electrode.^20^ The changes caused by DNA immobilization and hybridization were detected by directly monitoring the differential pulse voltammetric (DPV) response. Compared with other biosensors, such a sensor is quite convenient, safe and cheap because there is no fluorophore labelling or enzyme amplification step, and easy to fabricate. In addition, the high sensitivity is also a remarkable advantage.

## 2. Experimental section

### 2.1. Synthesis of GQDs by ‘bottom-up’ methods

The GQDs were synthesized by pyrolyzing citric acid (CA) based on the previous report,^17^ get 2 g of citric acid into a beaker, then heated to 200 °C and last for 20 min to get CA melt and pyrolyzed. From the color of the liquid, we can judge whether the formation of GQDs or not. Keep heating until the color of the liquid turned from colorless to faint yellow. Then, dissolved the liquid into 50 mL of 0.25 mol L^−1^ NaOH solutions and continuous stirring for about 30 min. At last, 0.25 mol L^−1^ NaOH solution was used to obtained faintly acid GQD solution.

### 2.2. Characterization of GQDs

Transmission electron microscopy (TEM) was performed with the H-9000 (JEOL Ltd. Japan), and high-resolution transmission electron microscopy (HRTEM) measurements was performed with JEM-2100F (JEOL Ltd. Japan) transmission electron microscopes. X-ray photo electron spectroscopy (XPS) was obtained with aKratos Axis Ultra-DLD XPS System (Kratos Analytical Ltd., Japan). Fourier transform infrared (FTIR) Spectra were measured with an FTIR spectrophotometer (Thermo Scientific Nicolet iN 10MX).

### 2.3. Preparation of GQDs modified GCE

We use 0.3 and 0.05 μm alumina slurries to polish the GCE sequentially. And then went through ultrasonic processing with ethyl alcohol and deionized water for 1 min, respectively. Drip 20.0 μL of the GQDs solution on the polished GCE surface and dried overnight at the room temperature to obtain a uniform film. The GQD modified GCE electrode was finally prepared by thoroughly rinsed with pure water. For comparison, the GCE without GQDs was also prepared and tested.

### 2.4. Immobilization and hybridization of DNA

**Fig. 1:**
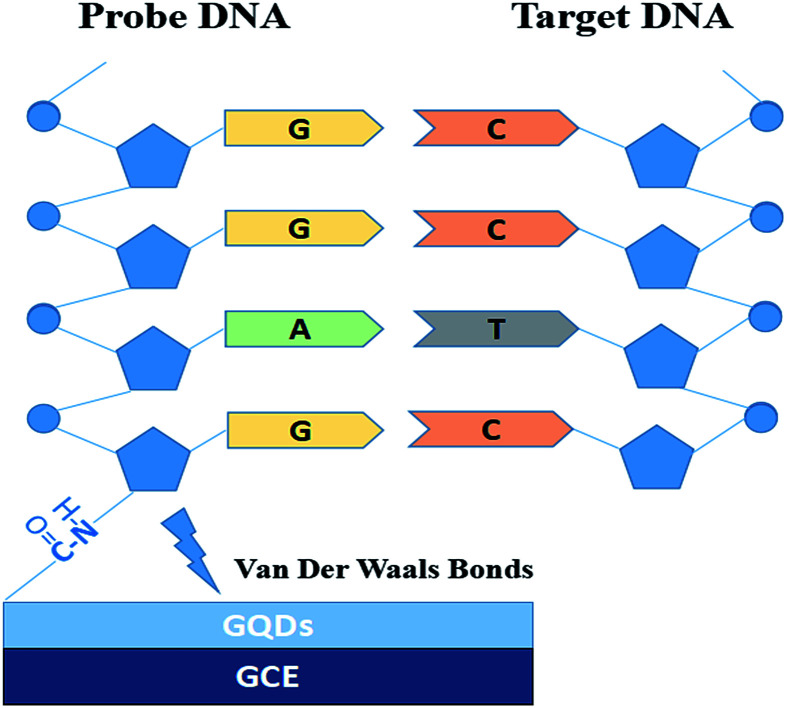
Electrode setup figure for DNA detection.

Fig. 1 is the electrode setup for DNA detection.

The probe DNA (pDNA) was immobilized onto the surface of the GQD modified electrode by the following method, 10 μL probe DNA (pDNA) solution (pH 7.0, containing 5.0 × 10^−7^ M pDNA) was dripped on the GQDs modified GCE surface and dried in a drying oven at 57 °C for 30 min since 57 °C is the recommended hybridization temperature in the oligo product information offered by the Shanghai Sangon Biological Engineering Technological company. After pDNA immobilization, dripping 20.0 μL complementary HBV-DNA (tDNA) on the pDNA modified GCE surface and similarly dried in the drying oven at 57 °C for 30 min to hybridize the pDNA and the tDNA. Then keep the electrode in 1.0 M KCl solution which contained 0.2 M K_3_[Fe(CN)_6_] at −0.7 V for 300 s to release the double-stranded DNA (dsDNA) which is produced by hybridization of pDNA and tDNA.^21–24^ Then electrochemical measurements were started on the electrode.

In this paper, we used the DNA oligonucleotides which are synthesized by Shanghai Sangon Biological Engineering Technological Co. Ltd (Shanghai, China). And in this experiment, the HBV-DNA base sequences we used are as below:

Probe DNA (pDNA): 5′-NH2-GAGGAGTTGGGGGAGGAGATT-3′; complementary DNA (tDNA): 5′-CTCCTCAACCCCCTCCTCTAA-3′; one-mid-base mismatch ssDNA (1MTDNA): 5′-CTCCTCAACCACCTCCTCTAA-3′; non-complementary ssDNA (ncDNA): 5′-ACTGCTAGATTTTCCACAT-3′.

### 2.5. Electrochemical measurements

The machine we used to perform electrochemical measurements was a CHI 760E electrochemical workstation (Shanghai CH Instrument Company, China) which had a conventional three-electrode system. A reference electrode (SCE) was the saturated calomel, the auxiliary electrode was a platinum wire and the GQDs modified GCE as the working electrode.

Cyclic voltammetry (CV) experiments were taken at a scan rate of 0.10 V s^−1^ from 0.8 V to −0.2 V and recorded in KCl solution which contained 0.2 M K_3_[Fe(CN)_6_]. Also, in the same solution, we made differential pulse voltammetry (DPV) experiments with a pulse width of 0.05 s, a pulse period of 0.5 s, and a pulse amplitude of 0.05 V. Before DPV scanning, the electrode underwent a process of preconditioning at −0.7 V for 300 s. All experiments mentioned above, were carried out under the room temperature.

## 3. Results and discussion

The GQDs were obtained by ‘bottom-up’ methods which carbonized CA at an appropriate degree. During the pyrolysis process of CA, the CA molecules self-assembled *via* the inter-molecular hydrogen bond and dehydration reaction, and then formed nanocrystalline GQDs with abundant –OH, C=O and –COOH groups on the edge.

**Fig. 2:**
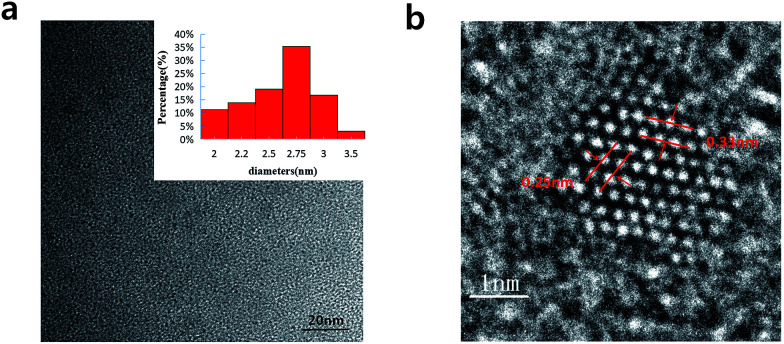
(a) TEM images of the GQDs. Inset images are size distributions (upper), (b) HRTEM image of the GQDs.

In Fig. 2a, we can see the TEM image with the low magnification and the corresponding size distribution histogram of GQDs. The as-prepared GQDs are with mean diameters of 2.6 nm and are well dispersed in narrow size distributions. In Fig. 2b, we can see a representative high resolution TEM image of an individual GQDs. The distinct crystal lattice indicates the crystallinity of the GQD, and the lattice parameter of 0.33 and 0.25 nm represents the (002) and (1120) lattice fringe of graphene, respectively.

**Fig. 3:**
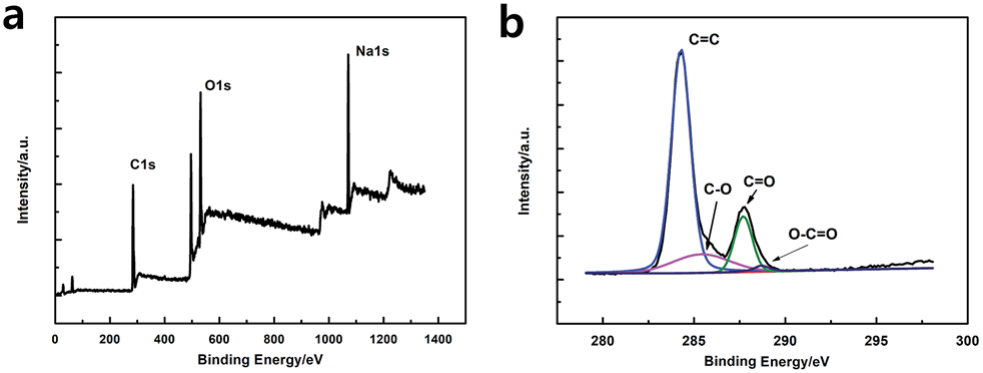
(a) XPS survey spectrum of GQDs. (b) High resolution C1s XPS spectra of GQDs.

As shown in Fig. 3a, the XPS spectra illustrates that the GQDs mainly consist of carbon (at *ca.* 284.8 eV) and oxygen (at *ca.* 531.4 eV). As shown in C1s high-resolution XPS spectra of GQDs (Fig. 3b), large number of oxygen functional groups can be found, which indicated the incomplete carbonization during the pyrolysis of citric acid.^17^ Note that C=O was the main oxygen functional groups, which was formed by CA molecules self-assembled and carboxyl group on the edges of GQDs.

**Fig. 4:**
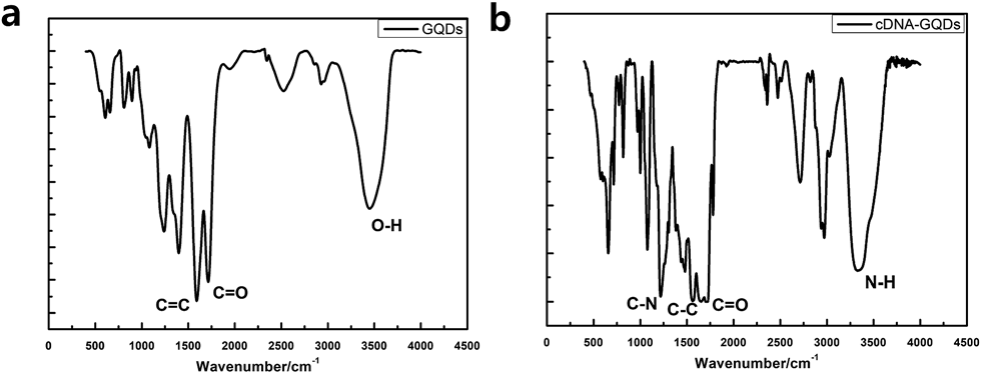
FT-IR spectra of the GQDs (a) and pDNA-GQDs (b).

To check whether the probe DNA can be immobilized onto the GQDs, FT-IR spectra were also measured to confirm the surface functional groups of GQDs and pDNA-GQDs (Fig. 4). As shown in the FT-IR spectrum of GQDs, the absorption band at 3460 and 1721 cm^−1^ were attributed to the stretching vibration of O–H and C=O, suggesting incomplete carbonization during the pyrolysis of citric acid and the carboxyl functional groups remained at the edge of GQDs. After adding probe DNA (pDNA) to the GQDs solution, the N–H bond and the C=O bond of the amide were found, and the C=O of carboxy absorption bands were weakened, which is attributed to the condensation reaction between the amino group of cDNA and the carboxyl group of GQDs, indicating that pDNA have been successfully modified on GQDs surface.

**Fig. 5:**
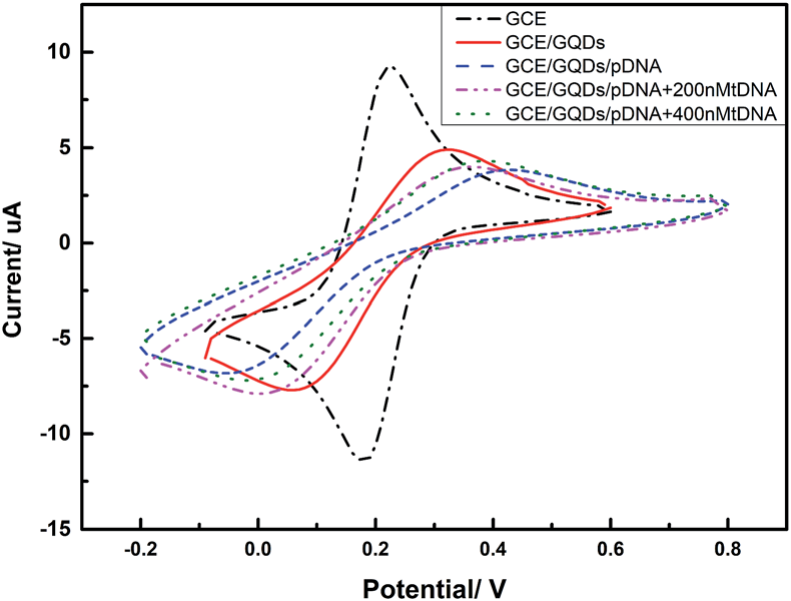
CVs of different electrodes (GCE, GQDs modified GCE, pDNA immobilized GQDs modified GCE, pDNA immobilized GQDs modified GCE with 200 nM tDNA, pDNA immobilized GQDs modified GCE with 400 nM tDNA) in a 1.0 M KCl solution containing 0.2 M K_3_[Fe(CN)_6_] solution.

We evaluated the feasibility of combining the GQDs and GCE as an electrochemical sensing platform by using K_3_[Fe(CN)_6_] as the electrochemical active species. The cyclic voltammetry (CV) was carried out in above KCl solution which contained 0.2 M K_3_[Fe(CN)_6_] solution and was used to characterize the modification of the GCE and the DNA fixation on the modified GCE. Fig. 5 shows the CV curves of GQDs modified GCE, bare GCE, and 100 μM probe DNA immobilized GQDs/GCE before and after adding tDNA, which demonstrates that the GQDs were absorbed on the surface of the GCE, and the pDNA was immobilized onto the surface of the GQD modified electrode successfully. The electron transfer between the electrode and the electro-active species K_3_[Fe(CN)_6_] will be inhibited due to the electrostatic repulsion which is caused by bind of immobilized pDNA and GQD film on the GCE electrode surface.^25^ Thus, we can observe an evidently decreased peak current of K_3_[Fe(CN)_6_] obtained at the pDNA–GQD modified electrode. However, after adding the target HBV-DNA (tDNA), the probe DNA will bind with the target instead of GQDs. For instance, the electrostatic repulsion to the electro-active species K_3_[Fe(CN)_6_] resulted from the immobilized pDNA will be removed. As a result, the obtained peak currents of K_3_[Fe(CN)_6_] will have a recovery with the different concentration of the target HBV-DNA.

**Fig. 6:**
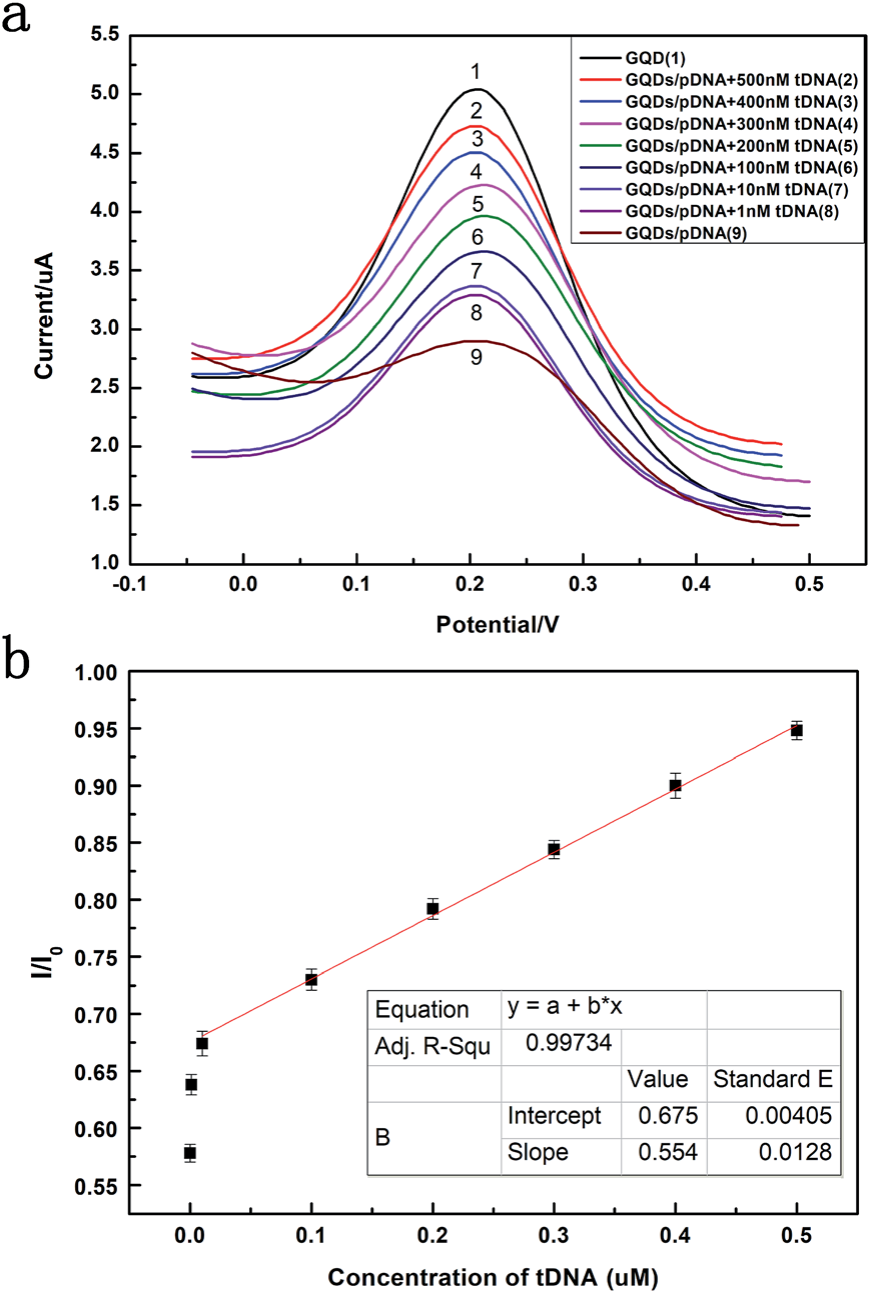
(a) Differential pulse voltammograms plots of 0.2 M K_3_[Fe(CN)_6_] at pDNA (500 nM) immobilized GQDs modified GCE in 1.0 M KCl and that after hybridization with different concentrations of HBV-DNA (tDNA). (b) The plot of the peak current against the concentration of HBV-DNA.

Fig. 6 exhibits the comparison of DPV curves. It shows the DPV signals of the GQDs modified GCE after different concentration (1 nM to 500 nM, 10 μL) HBV-DNA hybridizing with the pDNA. The DPV experiment was made in the above KCl solution which contains 0.2 M K_3_[Fe(CN)_6_]. As shown in Fig. 6a, when the concentration of probe DNA is fixed (500 nM), the peak current of K_3_[Fe(CN)_6_] obtained at the modified electrode increases along with the concentration of HBV-DNA, which illustrates that the amount of remaining pDNA had decreased, because the dsDNA formed by the hybridization of pDNA and tDNA had been released from the electrode surface by the electrochemical pretreatment (−0.7 V, 300 s). The more HBV-DNA molecules are in the test solution, the more dsDNA formed and escaped from the GQDs modified electrode, thus higher electrochemical response can be observed. The linear relationship between the peak current and the concentration of HBV-DNA was shown in Fig. 6b. As the plot of 1 nM deflected too much, the linear relationship used the data from 10 nM to 500 nM. It could be expressed as *y* = 0.55*x* + 0.67, where *R*^2^ = 0.99. The established method for HBV-DNA detection has a broad linear range of 10–500 nM with a detection limit of 1 nM DNA. And the limit of quantification (LOQ) is 10 nM.

**Fig. 7:**
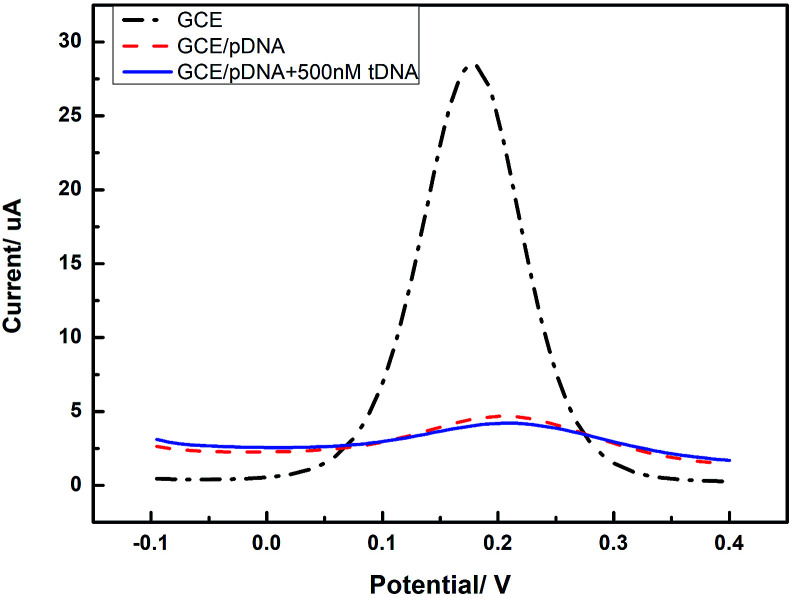
Differential pulse voltammetry obtained at an unmodified GCE electrode, the unmodified electrode further immobilized with pDNA, and the cases that pDNA has been incubated with HBV-DNA (tDNA).

In order to confirm the role of GQDs, we tested the DPV curves based on the GCE without the GQDs modified. As we can see in Fig. 7, after adding HBV-DNA, there is no increase of the peak currents of K_3_[Fe(CN)_6_]. Compared with the DPV curves based on the GQDs modified GCE, we can know that GQDs can immobilized DNA and allow double-stranded DNA to escape from electrode in time. Without GQDs modified, bare GCE could not effectively adsorb probe DNA and detect HBV-DNA.

**Fig. 8:**
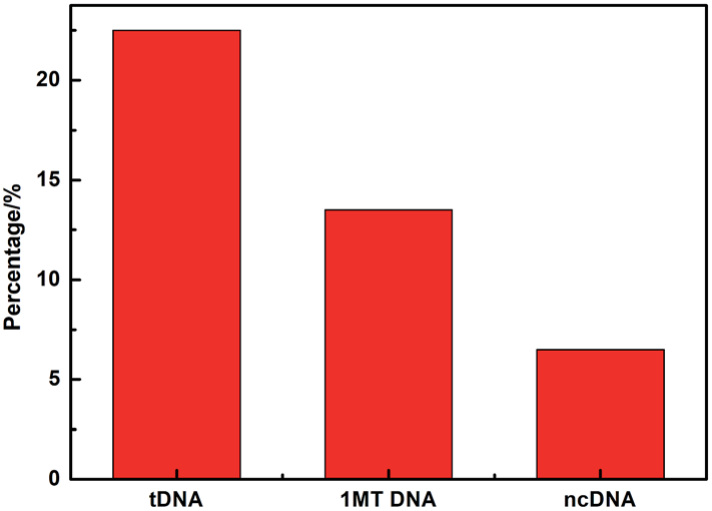
Comparison of hybridization signal changes for pDNA immobilized GQDs modified GCE with tDNA, single-base mismatched DNA (1MT DNA) and non-complementary DNA (ncDNA). The detection concentrations of tDNA, 1MT DNA and ncDNA were selected as 10 nM, 10 nM and 1 μM, respectively.

Fig. 8 compares three percentages of ΔIpc/Ipc in different ssDNA hybridization with the probe DNA, including the target complementary DNA (tDNA), one-middle-base mismatched DNA (1MT DNA), and non-complementary DNA (ncDNA) sequence. In order to simulate the real situation, this selectivity experiment was based on the actual situation of the blood in the ratio of the target complementary DNA and non-complementary DNA sequence. From the figure we can see that the highest ΔIpc is the complementary DNA sequence followed by 1MT DNA and the non-complementary DNA successively. Compared with the ΔIpc of the complementary DNA, the signal increasement of the non-complementary DNA was much lower and could be neglected. From this result we can conclude that the specificity of the DNA sensor is high selectivity and could distinguish most mismatched DNA sequences.

This DNA detection approach possesses a superior linear range and detection limit which is remarkable compared with that of relative previously report. As shown in Table 1, these results show that the GQDs modified GCE has an ultrahigh sensitivity for the DNA biosensing.

**Table 1:** GQDs based biosensor performance for the detection of DNA

Material	Preparation method	Detection method	Detection target	Linear range	Detection limit	Ref.
GQDs	Hydrothermal method	Electrochemistry	DNA sequence	200–500 nM	100 nM	25
GQDs	Hydrothermal method	Electrochemical luminescence	AuNPs-ssDNA	25–400 nM	13 nM	26
Carbon nanoparticles	—	Fluorescence	HIV-DNA	1–50 nM	0.4 nM	27
Streptavidin modified nanoparticles	—	Electrochemistry	HBV-DNA	—	2530 nM	28
Carbon paste	—	Electrochemistry	HBV-DNA	—	15 nM	3
GQDs	Pyrolysis method	Electrochemistry	HBV-DNA	10–500 nM	1 nM	This study

## 4. Conclusions

In summary, a new ultra-sensitive and effective label-free electrochemical HBV-DNA sensing platform has been established based on the prepared GQDs. The material was made by pyrolysis method, which is quite convenient, safe and cheap. The limit of detection could get 1 nM and detection range is from the 10 nM to 500 nM. It provides a universal method which can be used for DNA detection. This sensing system can distinguish complementary and mismatched nucleic acid sequences with high sensitivity and good reproducibility. Also, the proposed sensor could be used in detecting other DNA. Since all the materials involved in the sensing system are of excellent biocompatibility, it is expected that this DNA detection method would be used *in vitro* and can be extended for the detection of more molecules.

## Conflicts of interest

There are no conflicts to declare.

## Supplementary Material
